# The Influence of As-Cast Grain Size on the Formation of Recrystallized Grains and the Related Mechanical Properties in Al–Zn–Mg–Cu-Based Alloy Sheets

**DOI:** 10.3390/ma17215267

**Published:** 2024-10-29

**Authors:** Jonggyu Jeon, Sangjun Lee, Jeheon Jeon, Maru Kang, Heon Kang

**Affiliations:** 1 Department of Materials Science and Engineering, Yonsei University, Seoul 03722, Republic of Korea; garuda2927@yonsei.ac.kr (J.J.); hlqwe456@yonsei.ac.kr (S.L.); jeheon@lgensol.com (J.J.); 2 Institute of Industrial Science, The University of Tokyo, 4-6-1 Komaba, Meguro, Tokyo 153-8505, Japan; 3 Smart Agricultural Machinery R&D Group, Korea Institute of Industrial Technology, Cheonan 54325, Republic of Korea; mrkang@kitech.re.kr; 4 Customized Manufacturing R&D Department, Korea Institute of Industrial Technology, Siheung 15014, Republic of Korea

**Keywords:** Al–Zn–Mg–Cu alloys, grain refinement, recrystallization behavior, microstructure, mechanical properties

## Abstract

The influence of as-cast grain size on recrystallization and the related mechanical properties of Al–Zn–Mg–Cu-based alloys was investigated. Grain sizes ranging from 163 to 26 μm were achieved by adding Ti, Cr and Mn, and ZnO nano-particles, which acted as heterogeneous nucleation sites. A decrease in the as-cast grain size led to a corresponding reduction in the recrystallized grain size from 54 to 13 μm. Notably, as-cast grain sizes below 100 μm provided additional nucleation sites at grain boundaries, allowing for a reasonable prediction of recrystallized grain size. Finer grains also contributed to enhanced mechanical properties, with yield strength increasing as recrystallized grain size decreased without significant loss of elongation. Additional strengthening was observed due to η-precipitates at grain boundaries, further improving the properties of fine-grained sheets.

## 1. Introduction

Al–Zn–Mg–Cu-based alloys (7xxx series) are widely used in the automotive and aerospace industries because of their high strength-to-weight ratios, excellent toughness, and good machinability [1]. However, their properties must be improved to realize their potential for safety, lightness, and fuel efficiency and to extend the application of Al–Zn–Mg–Cu-based alloys [2]. The effective methods for improving the mechanical properties of Al–Zn–Mg–Cu-based alloys are as follows: precipitation and grain boundary strengthening. Increasing the contents of the major alloy components, such as Zn, Mg, and Cu, is effective for improving the strength but significantly reduces the toughness [3]. However, grain refinement can simultaneously improve strength and toughness and has constantly attracted research interest over the past decades.

The typical method for fabricating Al–Zn–Mg–Cu alloy sheets with a significantly reduced grain size is to control the thermomechanical treatment (TMT) conditions. The advanced TMT processes involve multi-step heat treatment and deformation to control nano-scale and micro-scale particles and can reduce the recrystallized grain size to less than 20 μm [4,5,6,7]. The finely dispersed nano-scale particles (<500 nm) inhibit the movement of grain boundaries (known as the Zener pinning effect) and delay recrystallization, resulting in a large recrystallized grain size [4,8]. The micro-scale particles (>1 μm) induce particle-stimulated nucleation (PSN), a phenomenon in which highly strained regions near the particles provide preferred nucleation sites for recrystallization, reducing the recrystallized grain size [5]. So far, numerous studies on Al–Zn–Mg–Cu-based alloy sheets have been conducted to find optimal conditions for heat treatment to form micro-scale particles suitable for PSN. Huo et al. [6] reported that an AA7075 sheet with a recrystallized grain size of approximately 10 μm was obtained by performing over-aging at 400 °C for 8 h before warm rolling. According to the published articles of Smolej et al. [7], AA7475 sheets with a recrystallized grain size of approximately 10 μm were fabricated via two-step heat treatment (at 485 °C for 4 h + at 400 °C for 8 h). However, the complicated process conditions of the advanced TMT processes require high operating costs and energy consumption.

Importantly, the as-cast grain boundaries have also been found to be potential nucleation sites for recrystallization because they have a large orientation gradient in a manner similar to the principle occurring near micro-scale particles [8,9,10,11,12,13,14]. The nucleation of recrystallization is generally associated with the regions where high dislocation density and large misorientations with respect to the surrounding matrix develop during deformation [15,16]. The slip system of dislocations changes near the as-cast grain boundaries, causing accumulation of dislocations. Thus, the static recrystallization can begin in the regions with a large orientation gradient that exist near the as-cast grain boundaries. In addition, dynamically recrystallized grains nucleate almost exclusively at high-angle grain boundaries during deformation [17,18]. Marthinsen [11] simulated the size distribution of recrystallized grains when recrystallization was repeated in both one- and two-dimensional microstructures. The results indicated that repeated recrystallization was performed at each step using the previously recrystallized grain structure, such as the grain boundaries and grain boundary corners, as nucleation sites. That is, even when the conventional TMT processes were performed, an Al–Zn–Mg–Cu alloy sheet with fine recrystallized grains could be produced by using the as-cast grain boundaries as nucleation sites for recrystallization. Bay et al. [19] reported that the number of potential nucleation sites for recrystallized grains, such as grain boundaries and deformation bands, is larger in fine-grained cast alloys than in coarse-grained cast alloys. According to the report of Roy [20], when a similar number of nucleation sites are formed in fine- and coarse-grained cast alloys, the fine-grained cast alloys exhibit more uniform recrystallization behavior. According to the aforementioned relationship, the recrystallized grain size decreased as the average grain size in the cast alloy prior to deformation decreased. However, the formation behavior of recrystallized grains in the Al–Zn–Mg–Cu alloy sheets has not yet been systematically studied with respect to the variation in the as-cast grain size.

In this study, for Al–Zn–Mg–Cu-based alloys, the influence of as-cast grain size on the formation of recrystallized grains was investigated. The cast alloys with different grain sizes ranging from 163 to 26 μm were prepared by cumulatively adding Ti, Cr and Mn, and ZnO nano-particles. During the conventional TMT processes, an Al–Zn–Mg–Cu recrystallized sheet with a grain size of 13 μm was fabricated by using the as-cast grain boundaries as preferred nucleation sites for recrystallization. The formation behavior of recrystallized grains was systematically studied with respect to the variation in the as-cast grain size. Furthermore, the mechanical properties of the recrystallized and aged sheets with fine grains were evaluated and their strengthening mechanisms were discussed.

## 2. Experimental Procedures

### 2.1. Preparation of Materials

**Figure 1:**
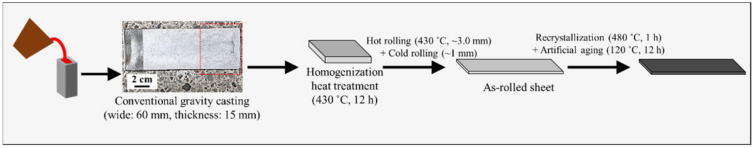
Schematic of the experimental procedure.

The alloys were fabricated using pure Al (99.8% Al), Zn (99.9% Zn), Mg (99.8% Mg), Cu (99.9% Cu), and master alloys (in wt.%), such as Al-40Cr, Al-20Mn, and Al-10Ti. The pure Al was placed in a graphite-coated SiC crucible and melted in a furnace at 760 °C. When transition metal elements, such as Ti, Cr, and Mn, were added, the melting temperature was raised to 820 °C, maintained for 1 h, and then lowered to 760 °C. Then, the other alloy components were added to the melt, and the temperature was maintained for a suitable time to completely dissolve all alloy elements. ZnO nano-particles (1.0 wt.%) with a mean diameter of 20 nm were added to the melt, and mixing was performed using a graphite stick. This mixture was maintained for 1 h to induce a reaction between Al and ZnO nano-particles. The melt was cast into a steel mold preheated at 300 °C with a thickness of 15 mm and then quenched in water (Figure 1). Table 1 presents the chemical compositions of the alloys, which were determined using optical emission spectrometry (SPECTROCHECK, Ametek Co., USA).

The cast alloys (A, B, C, and D) were rolled after homogenization heat treatment at 430 °C for 12 h in an air-circulation furnace. The rolling process was performed in two stages: hot rolling from 15 to 3 mm at 430 °C, followed by cold rolling to 1 mm, with a reduction of 20%. The cold-rolled sheets were recrystallized at 480 °C for 1 h in an air-circulation furnace, followed by water quenching. Artificial aging was performed at 120 °C for 12 h in an air-circulation oven.

### 2.2. Evaluation of Mechanical Properties

The mechanical properties of the alloy sheets were evaluated using the yield strength (YS, 0.2% offset flow stress), ultimate tensile strength (UTS), and elongation (EL). Tensile tests were performed using an Instron-type machine with a strain rate of 10^−3^ s^−1^ at 25 °C. Tensile specimens (gauge length: 25.4 mm; width: 6.35 mm; thickness: 1 mm) in compliance with the ASTM E8 standard were machined from the Al sheets.

### 2.3. Characterization of Microstructures

The microstructures of the cast alloys and alloy sheets were examined using optical microscopy (OM, Nikon Eclipse LV150, Japan) and scanning electron microscopy (SEM, JEOL JSM-7001F, Japan). The specimens used for grain size observation were prepared by etching with Keller’s reagent (95% H_2_O, 2.5% HNO_3_, 1.5% HCl, and 1.0% HF by volume). High-resolution transmission electron microscopy (TEM, FEI Titan^TM^ 80-300, USA) was performed at 300 kV, and a chemical analysis was performed using energy-dispersive X-ray spectroscopy (EDS). The specimens for the TEM analysis were prepared using mechanical thinning followed by ion milling (Gatan Model 691,USA). The crystal orientations of the recrystallized sheets were observed using electron backscatter diffraction (EBSD, EDAX-TSL, USA) together with SEM. The specimens for the EBSD analysis were mechanically polished, and then the surface strain was eliminated by ion milling prior to the EBSD observations. The inverse pole figure (IPF) maps were acquired 15% above the surface with a step size of 2.0 μm at ×200 magnification. The orientation distribution function (ODF) was obtained using the SL-OIM analysis software. The Kernel average misorientation (KAM) distributions indicated the average misorientation angle of a given point with respect to all its third neighbors. KAM maps of sheets deformed by 20% were obtained from IPF maps acquired with a step size of 1 μm at ×400 magnification. The sheets deformed by 20% were prepared using the Instron-type machine: the sheets were tensioned at a strain rate of 10^−3^ s^−1^, followed by termination at 20% engineering strain. The average grain size was measured using the linear intercept method (ASTM: E-112 standard). The software ImageJ was used to calculate the size and volume fraction of the second-phase particles.

## 3. Experimental Results

### 3.1. Microstructure Evolution of Cast Alloys

**Figure 2:**
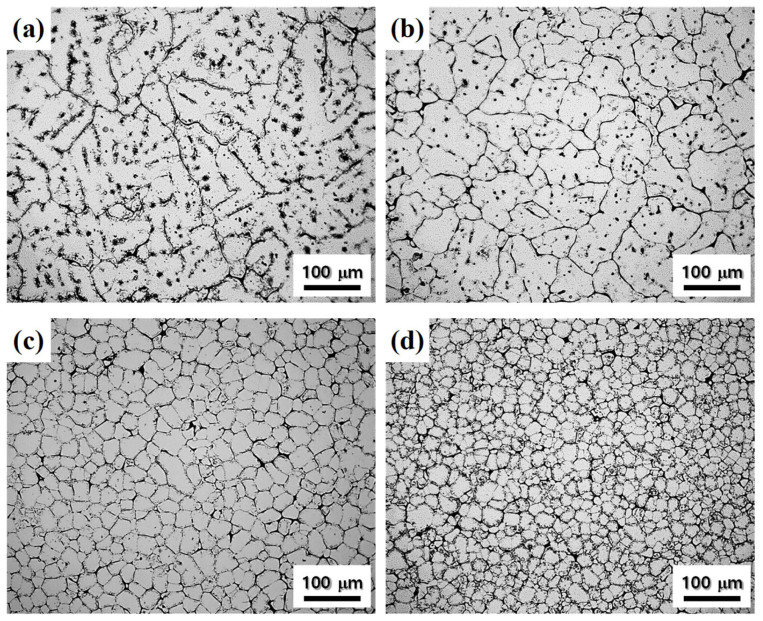
OM images of the as-cast alloys: alloys (**a**) A, (**b**) B, (**c**) C, and (**d**) D. When the Ti, Cr and Mn, and ZnO nano-particles are added cumulatively, the average grain size of cast alloys decreases from 163 to 26 μm.

OM images of cast alloys are shown in Figure 2. The average grain size of cast alloys A, B, C, and D is 163, 87, 43, and 26 μm, respectively. The average grain size of cast alloy B is reduced to 87 μm by adding 0.18 wt.% Ti, and the average grain size of cast alloy C is reduced to 43 μm by adding 0.04 wt.% Cr and 0.17 wt.% Mn. Alloy D with ZnO nano-particles added shows a grain size of 26 μm smaller than other cast alloys investigated in this study. The grain refinement mechanism of the cast alloys is discussed in Section 4.1.1.

**Figure 3:**
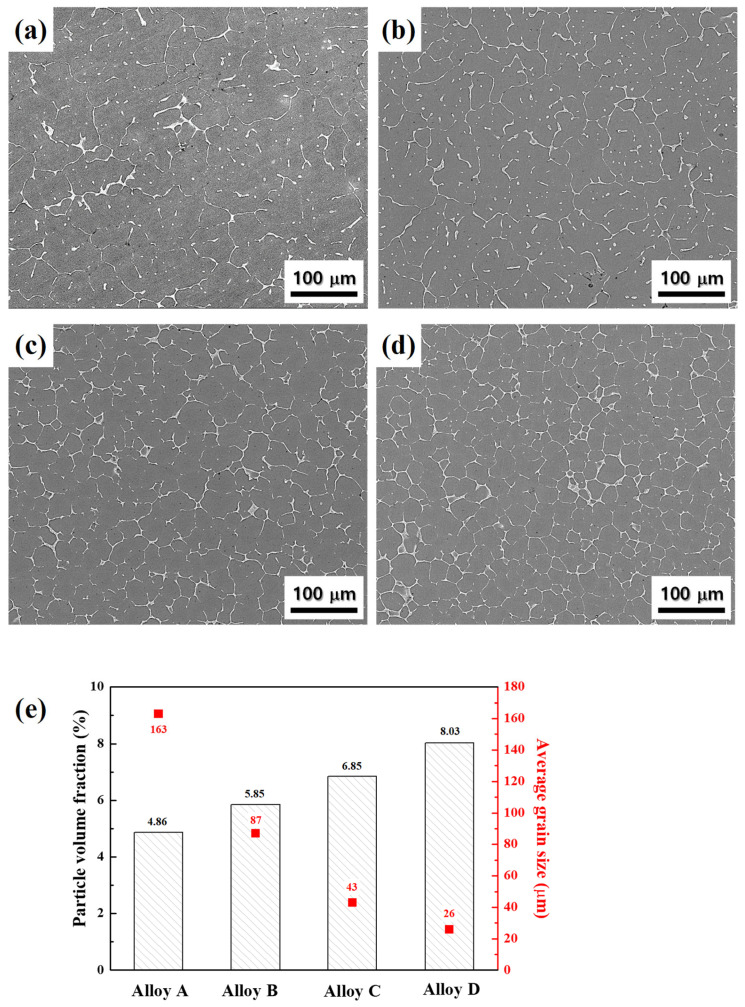
SEM images of the as-cast alloys: alloys (**a**) A, (**b**) B, (**c**) C, and (**d**) D. (**e**) The variation in the volume fraction of eutectic particles plotted with the average grain size of cast alloys. As the average grain size of the cast alloys decreases from 163 to 26 μm, the volume fraction of the eutectic particles increases from 4.86 to 8.03 vol.%.

SEM images of the cast alloys are shown in Figure 3a–d. During the non-equilibrium solidification process, solute atoms, such as Zn, Mg, and Cu, are segregated at grain boundaries and interdendritic interfaces, forming a T phase (Al_4_Mg_2_CuZn or Al_3_Mg_3_CuZn_2_) [21,22]. In alloys A and B with the grain sizes of 163 and 87 μm, the eutectic particles coexist in the grain boundaries and interdendritic interfaces. On the other hand, in alloys C and D with relatively small grain sizes of 43 and 26 μm, the eutectic particles preferentially exist at the grain boundaries, forming a network structure [23]. As shown in Figure 3e, as the average grain size of the cast alloys decreases from 163 to 26 μm, the volume fraction of the eutectic particles increases from 4.86 to 8.03 vol.%.

**Figure 4:**
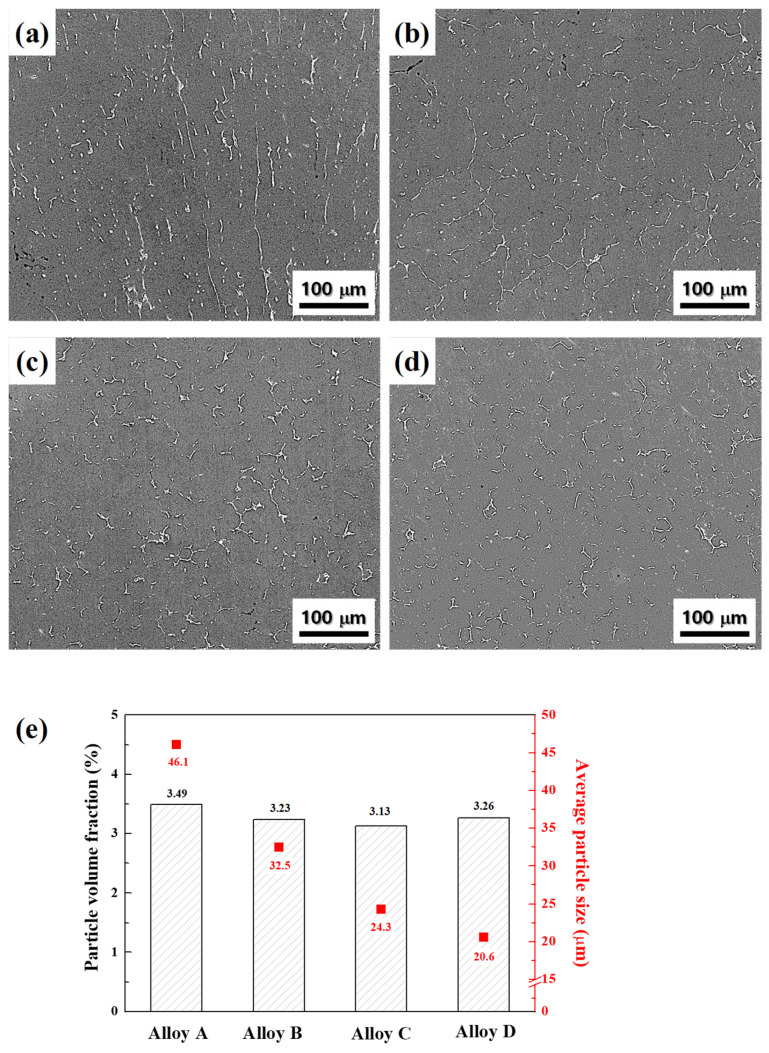
SEM images of alloys with heat treatment for homogenization at 430 °C for 12 h: alloys (**a**) A, (**b**) B, (**c**) C, and (**d**) D. (**e**) The variation in the particle size and volume fraction of eutectic particles with the average grain size of cast alloys. The average size of the remaining eutectic particles significantly reduces from 46.1 to 20.6 μm as the as-cast grain size decreases.

SEM images of alloys with heat treatment for homogenization at 430 °C for 12 h are shown in Figure 4a–d. The eutectic particles were partially dissolved in the Al matrix and left a large amount of residue. As shown in Figure 4e, the volume fraction of the eutectic particles in homogenized alloys A, B, C, and D was 3.49, 3.23, 3.13, and 3.26 vol.%, respectively. The volume fraction of the eutectic particles became similar without being affected by the as-cast grain size because they have similar contents of Zn, Mg, and Cu and the equilibrium concentration of solid solution. On the other hand, the average size of the remaining eutectic particles was significantly reduced from 46.1 to 20.6 μm, as the as-cast grain size decreased from 163 to 26 μm.

### 3.2. Microstructure Evolution of Alloy Sheets

**Figure 5:**
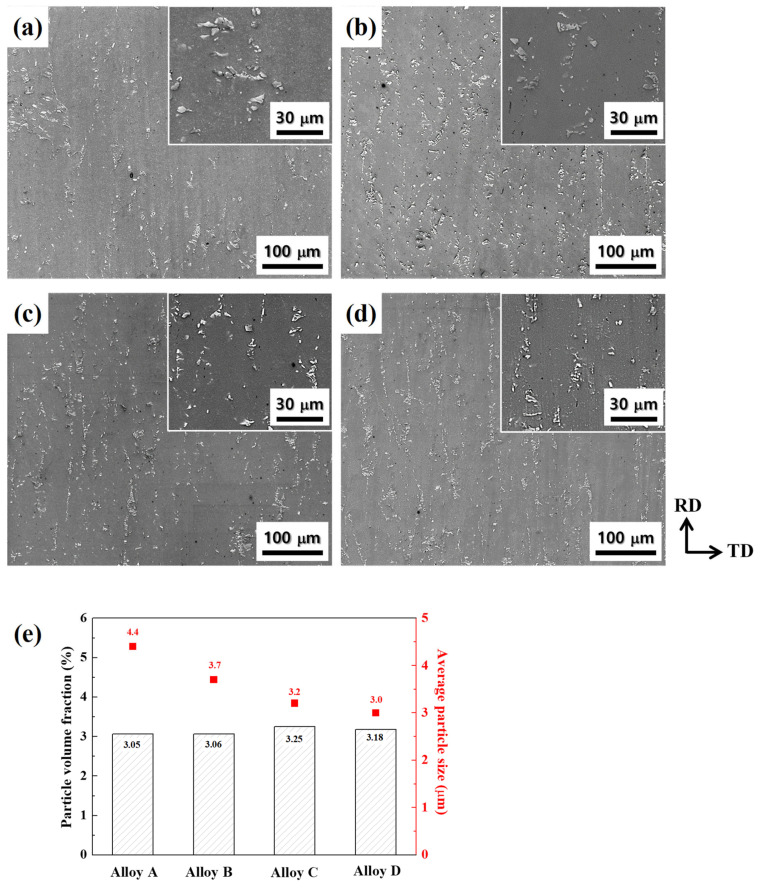
SEM images of hot-rolled sheets with thickness of 3 mm: alloy sheets (**a**) A, (**b**) B, (**c**) C, and (**d**) D. Enlarged images for each specimen are shown in the white rectangle. (**e**) The variation in the particle size and volume fraction of eutectic particles of the alloy sheets. The average size of eutectic particles decreases from 4.4 to 3.0 μm as the as-cast grain size decreases.

Figure 5a–d shows SEM images of the alloy sheets with a thickness of 3 mm before cold rolling. The eutectic particles were fractured and aligned in the rolling direction (RD). As shown in Figure 5e, the volume fractions of the eutectic particles in alloy sheets A, B, C, and D were 3.05, 3.06, 3.25, and 3.18 vol.%, respectively. The average size of eutectic particles in alloy sheets A, B, C, and D was 4.4, 3.7, 3.2, and 3.0 μm, respectively. As the average grain size of the cast alloys decreased, the fractured particle size also decreased.

**Figure 6:**
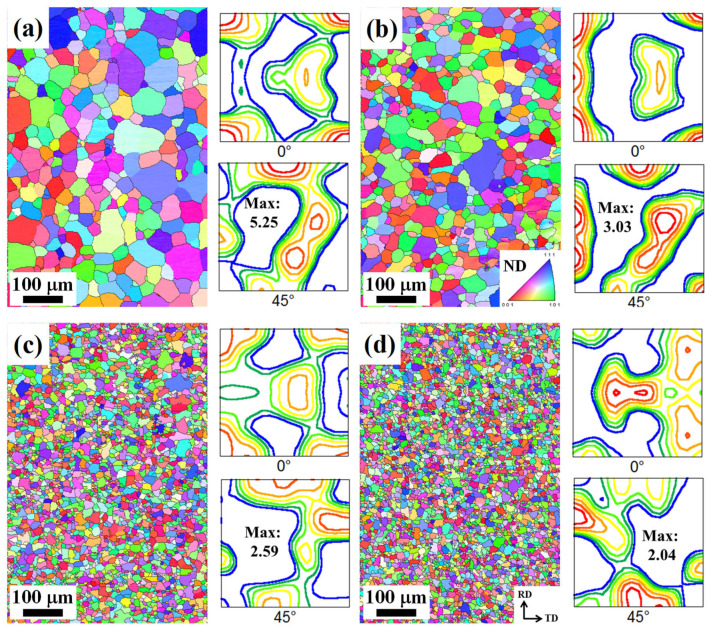
IPF maps and ODF of alloy sheets recrystallized at 480 °C for 1 h according to their crystallographic direction along the normal direction (ND) of the sheet: alloy sheets (**a**) A, (**b**) B, (**c**) C, and (**d**) D. The average grain size of alloy sheets A, B, C, and D is 54, 37, 16, and 13 μm, respectively. Maximum intensity decreased from 5.25 to 2.04 as the average grain size decreased from 54 to 13 μm.

Figure 6 shows the IPF maps and ODF of sheets recrystallized at 480 °C for 1 h. As the as-cast grain size decreased from 163 to 26 μm, the recrystallized grain size decreased correspondingly. The average grain size of recrystallized sheets A, B, C, and D was 54, 37, 16, and 13 μm, respectively. The grain refinement mechanism of the recrystallized sheets is discussed in Section 4.1.2. The maximum intensity of the texture components gradually decreased from 5.25 to 2.04 as the recrystallized grain size decreased from 54 to 13 μm, which indicates the formation of relatively random textures.

**Figure 7:**
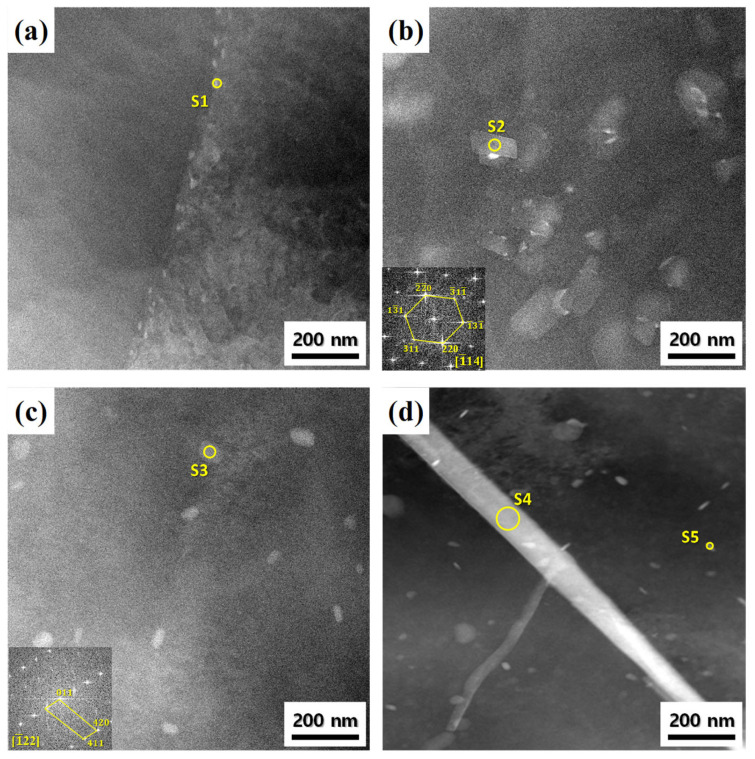
Scanning TEM images of the alloy sheets recrystallized at 480 °C for 1 h: alloy sheets (**a**) A, (**b**) B, (**c**) C, and (**d**) D. The chemical compositions of the marked particles from S1 to S5 are presented in Table 2.

The TEM images in Figure 7 show various nano-scale particles formed on the recrystallized sheets A, B, C, and D. The chemical compositions of the nano-scale particles labeled S1 to S5 are presented in Table 2. In sheet A, the η-precipitates with a size of approximately 15 nm were formed along the grain boundaries. In sheet B to which Ti was added, plate-shaped particles having a size of 100–150 nm were densely formed. The plate-shaped particles contained a relatively large amount of Mg and Ti and had a cubic structure with a lattice parameter of 1.482 nm. In sheet C with the addition of Cr and Mn, there were round- and rod-shaped particles with a size of approximately 50 nm, which had a cubic structure with a lattice parameter of 0.9119 nm. In sheet D to which ZnO nano-particles were added, large needle-shaped particles (several micrometers in length and 80 nm in width) and small needle-shaped particles (approximately 30 nm in length and 5 nm in width) were observed. The large and small needle-shaped particles contained large amounts of Zn, Mg, and Cu, but the small needle-shaped particles contained relatively large amounts of Zn. However, the developmental mechanism for the large and small needle-shaped particles would require further study.

### 3.3. Mechanical Properties of Alloy Sheets

**Figure 8:**
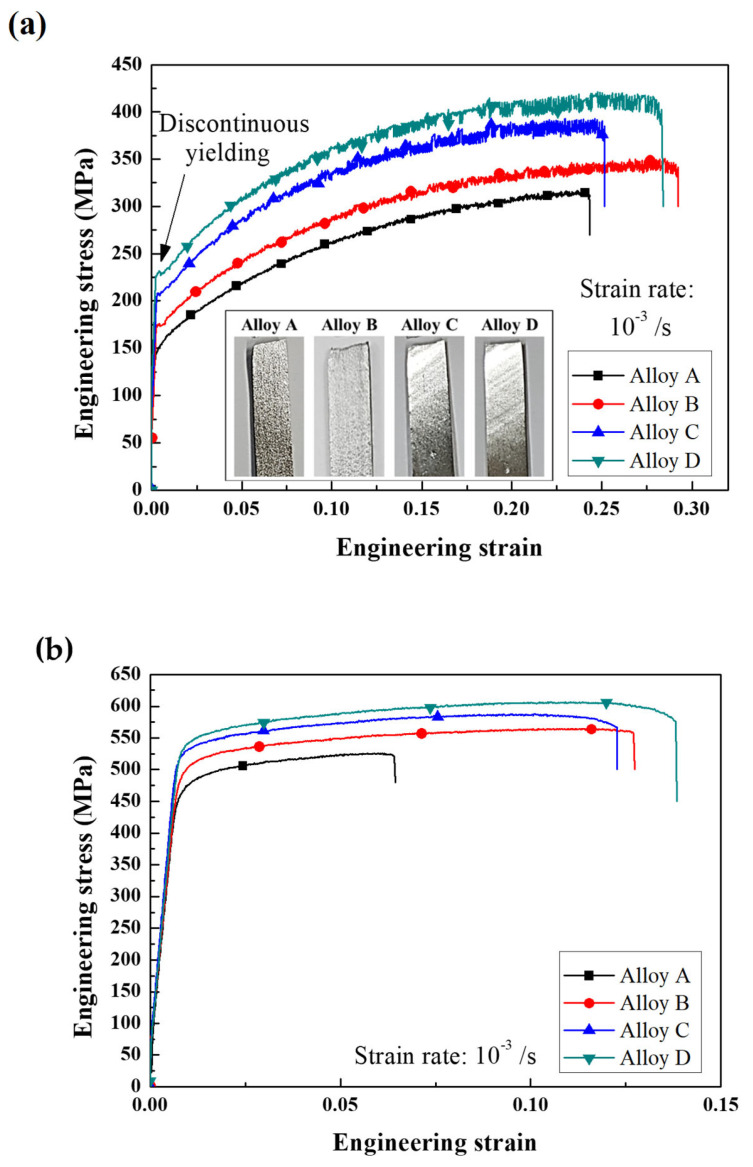
Engineering stress–strain curves of alloy sheets: (**a**) sheets recrystallized at 480 °C for 1 h and (**b**) sheets aged at 120 °C for 12 h. Surface images of the deformed tensile specimens were interpolated in (**a**). When Ti, Cr and Mn, and ZnO nano-particles are cumulatively added (as the average grain size of the alloy sheets decreases), the YS and UTS of recrystallized and aged sheets gradually increase without significant loss of the EL.

The engineering stress–strain curves of the recrystallized and aged sheets are shown in Figure 8. As Ti, Cr and Mn, and ZnO nano-particles were cumulatively added (as the average grain size of the sheets decreased), the YS and UTS of the recrystallized and aged sheets gradually increased without significant loss of the EL. As shown in Figure 8a, in alloy D, compared to alloy A, YS increased by approximately 78 MPa, and elongation increased by approximately 4%. Alloy sheets B, C, and D with an average grain size of <37 μm exhibited a discontinuous yielding phenomenon (the Piobert–Lüders effect), which is a phenomenon where the solute atoms, such as Zn and Mg, act as obstacles to the dislocation movement. This is because the mobile dislocations of fine-grained alloys encounter obstacles more frequently than those of coarse-grained alloys [24]. In addition, the surface roughness gradually decreased as the average grain size of the recrystallized sheets decreased. After the aging treatment (Figure 8b), the discontinuous yielding phenomenon disappeared because solute atoms were used to form η (MgZn_2_)-precipitates. Owing to the precipitation strengthening of the η, the YS significantly increased by approximately 250 MPa. The average grain size, YS, UTS, and EL of the recrystallized and aged sheets are summarized in Table 3. Details regarding the strengthening effect are discussed in Section 4.2.

### 3.4. Microstructure Evolution of Deformed Sheets

**Figure 9:**
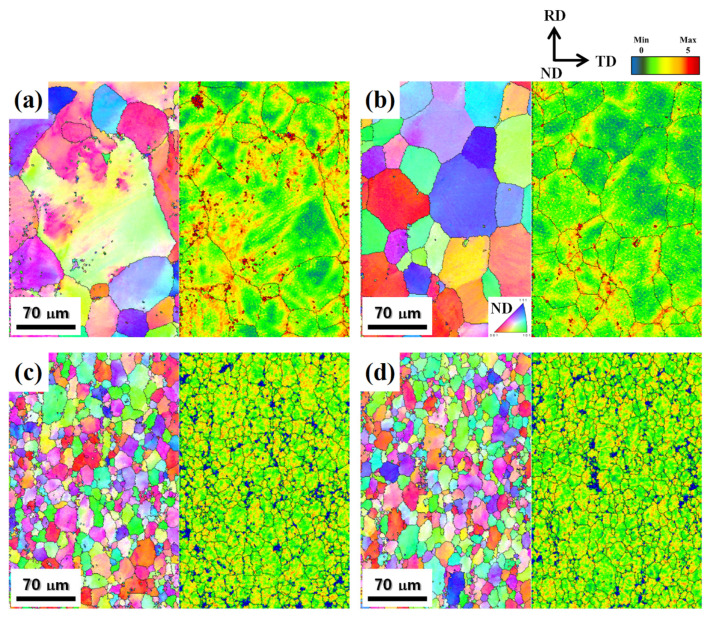
IPF and KAM maps of sheets deformed by 20%: alloy sheets (**a**) A, (**b**) B, (**c**) C, and (**d**) D. The average grain size of alloy sheets A, B, C, and D was 92, 55, 20, and 19 μm, respectively. The stored strain energy was concentrated at local grain boundaries in the alloy sheets A and B, whereas the stored strain energy was distributed uniformly over the entire grain boundary in the alloy sheets C and D.

Figure 9 shows the IPF and KAM maps of 20% deformed sheets at ×400 magnification. The average grain size of deformed sheets A, B, C, and D was 92, 55, 20, and 19 μm, respectively. After 20% deformation, the recrystallized grains were elongated in the deformation direction (parallel to the RD), and the grain growth occurred. This is because the grains rotated around the fixed loading axes, resulting in the alignment of grains with a low-angle boundary as one grain [25]. The KAM analysis only considers local misorientations of <5° to eliminate the effects of grain boundaries. A larger KAM value indicates a larger amount of stored strain energy or accumulated dislocations [26,27]. For alloy sheets A and B with large grains (Figure 9a,b), the stored strain energy was concentrated at local grain boundaries. On the other hand, for alloy sheets C and D with relatively small grains (Figure 9a,b), the stored strain energy was distributed uniformly over the entire grain boundary.

## 4. Discussion

### 4.1. Grain Refinement Mechanism

#### 4.1.1. Grain Refinement of Cast Alloys

In general, the Al–Ti master alloys, which typically contain Al_3_Ti particles and an extremely small amount of solid-solution Ti, have been used extensively as a grain refiner. The Al_3_Ti particles have a tetragonal structure (a = 0.3863 nm, c = 0.8587 nm [28]) and similar lattice parameters to Al (a = 0.4048 nm) with an error of approximately 4.6%, acting as heterogeneous nucleation sites for α-Al. Additionally, an Al melt with a Ti content higher than 0.15 wt.% is referred to as a hyper-peritectic region, and the Al_3_Ti particles are stable in the melt of these regions [29,30]. Therefore, in alloy B, to which 0.18 wt.% Ti was added, the average grain size of the cast alloy was reduced from 163 to 87 μm. Cr and Mn have a strong chemical affinity to Al because their d-electron shell is not full; thus, they easily enter the atomic clusters of Al_3_Ti particles. The atomic clusters containing Cr and Mn were more stable and grew easily, preventing Al_3_Ti particles from dissolution and acting as efficient nucleation sites [31]. Therefore, for alloy C to which 0.04 wt.% Cr and 0.17 wt.% Mn were added, the average grain size was reduced from 87 to 43 μm.

Shin et al. [32] and El-Wazery et al. [33] reported that ZnO nano-particles refine the grains of Al. Many researchers have examined the reaction conditions between Al and ZnO particles. The reaction between Al powder and micro-sized ZnO particles is initiated at 970 °C [34], and the ball-milling process can lower the reaction temperature to 563 °C [35]. These reaction conditions are determined by the chemical reactivity and the interfacial conditions between the Al and ZnO particles. In the present study, nano-sized ZnO particles were used to enhance the chemical reactivity according to the theory of the Gibbs–Thomson effect [36]. When the ZnO nano-particles were introduced into the Al melt at 760 °C, decomposition occurred with Zn and O atoms. As can be seen from Table 1, the relatively high content of Zn in alloy D is because the decomposed Zn atoms are dissolved in the Al matrix. However, the decomposed O atoms were expected to be present in two ways: dissolving in the melt and forming oxide particles [32]. The decomposed O atoms reacted with Mg to form MgO particles due to the high oxygen affinity of Mg, but it was difficult to find individual MgO particles in the microstructure observations. The MgO particles with a similar lattice parameter (a = 0.4212 nm) to Al (with an error of approximately 4.0%) provided heterogeneous nucleation sites. Thus, for alloy D to which 1.0 wt.% ZnO nano-particles were added, the average grain size was reduced from 43 to 26 μm.

#### 4.1.2. Grain Refinement of Recrystallized Sheets

The refining mechanism of recrystallized grains between coarse- and fine-grained cast alloys is shown in Figure 9. The recrystallized grain size is determined by the total number of nuclei induced by the micro-scale particles and as-cast grain boundaries. In the fine-grained cast alloys, the large area of the as-cast grain boundary provides numerous potential nucleation sites for the recrystallized grains.

The refinement effect of the recrystallized grains by PSN is determined by the size and volume fraction of micro-scale particles (>1 μm). As the particle size decreases and their volume fraction increases, the recrystallized grain size is significantly reduced due to the high efficiency of PSN [37,38]. More specifically, the efficiency of PSN can be explained through the interaction between the micro-scale particles and dislocations, known as the Orowan looping model [39]. When the particle size decreases and their volume fraction increases, the inter-particle spacing becomes narrower. The narrow inter-particle spacing induces a strong interaction between the particles and dislocations, causing the PSN to occur actively. The relationship between the recrystallized grain size ($D_{R}$) and the size ($d_{P}$) and volume fraction ($f_{P}$) of micro-scale particles in Al–Zn–Mg–Cu alloys can be defined as follows:

(1) $$D_{R}=0.5\times d_{P}/f_{P}-15.$$

The recrystallized grain size has a linear relationship with the ratio of size and volume fraction of the particles [38].

**Figure 10:**
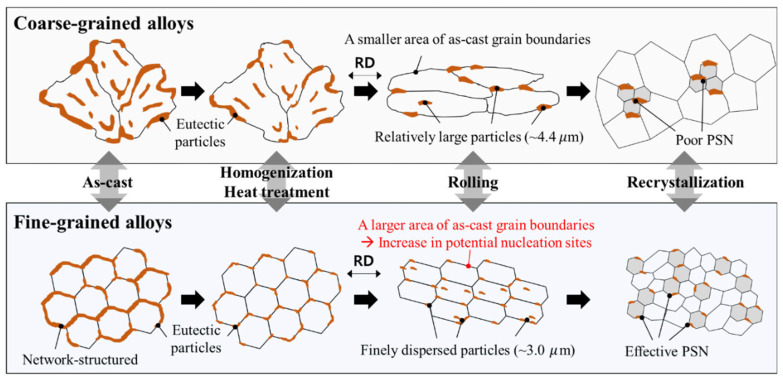
Schematic of the grain refinement mechanism during the conventional TMT processes in coarse- and fine-grained alloys. A larger area of as-cast grain boundary provides numerous potential nucleation sites for recrystallized grains, and the finely dispersed micro-scale particles induce active PSN.

In this study, the grain refinement effect induced by particles and grain boundaries is shown in Figure 10. The eutectic particle sizes in hot-rolled sheets A, B, C, and D were 4.4, 3.7, 3.2, and 3.0 μm, respectively, and their volume fractions of the eutectic particles were 3.05, 3.06, 3.25, and 3.18 vol.%, respectively (Figure 5e). Here, the $d_{P}/f_{P}$ values of 144.3, 120.9, 98.5, and 94.9 μm in alloy sheets A, B, C, and D, respectively, had no linear relationship with the recrystallized grain sizes of 54, 37, 16, and 13 μm. This is because not only the micro-scale particles, but the as-cast grain boundaries also greatly affect the formation of recrystallized grains. The grain refinement effect induced by the as-cast grain boundaries can be calculated by subtracting the measured grain size from the grain refinement effect induced by particles. For example, in the alloy sheet D, the grain refinement effect induced by the micro-scale particles and as-cast grain boundaries was approximately 24.6 and 19.5 μm, respectively. Consequently, as the as-cast grain size decreased, the number of nuclei contributed by the micro-scale particle and as-cast grain boundaries simultaneously increased, reducing the recrystallized grain size from 54 to 13 μm.

Importantly, the area of as-cast grain boundaries is proportional to the number of nuclei for the recrystallized grains induced by the grain boundaries. This is because the number of potential nucleation sites for recrystallized grains increases as the area of the as-cast grain boundaries increases. The area of as-cast grain boundaries per unit volume ($A_{G.B.}$) can be expressed as follows [40]:

(2) $$A_{G.B.}=6/D_{Cast},$$

where $D_{Cast}$ is the as-cast grain size. The grain shape was assumed to be a cube, which can physically be filled without an empty space, not a sphere. In addition, the number of grains is equal to the number of nuclei because every nucleus gives rise to one single grain [41]. Therefore, the total number of nuclei for recrystallized grains per unit volume ($N_{RX}$) can be calculated using the recrystallized grain size ($D_{RX}$) as follows [42]:

(3) $$N_{RX}=\frac{1}{{D_{RX}}^{3}},$$

where the $D_{RX}$ was measured in this study. The number of nuclei for the recrystallized grains induced by the grain boundaries ($N_{G.B.}$) can be expressed as follows:

(4) $$N_{G.B.}=N_{RX}-N_{Particle}.$$

**Figure 11:**
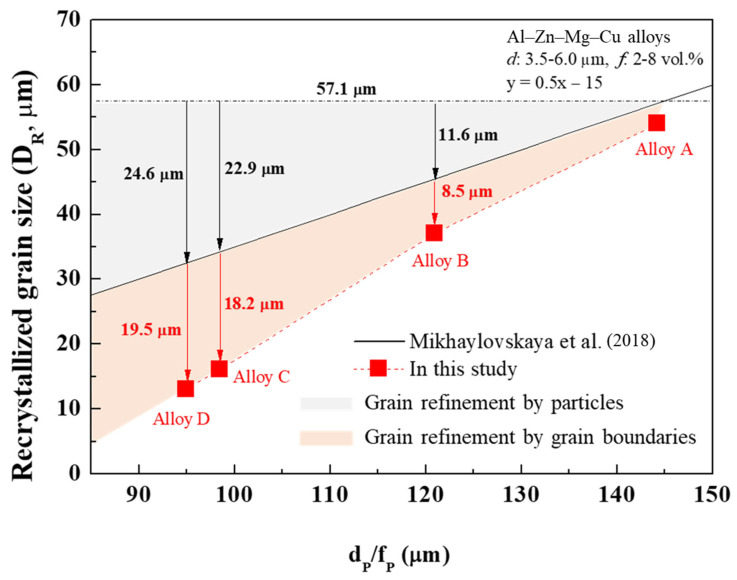
Recrystallized grain size vs. the ratio of the particle size to volume fraction ($d_{P}/f_{P}$). The effect of grain refinement by micro-scale particles is indicated by solid black line. Thus, the effect of grain refinement contributed by micro-scale particles and grain boundaries can be separated. The alloy A has little effect of grain refinement contributed by grain boundaries, whereas alloys C and D have a large effect.

$N_{Particle}$ is the number of nuclei for recrystallized grains induced by micro-scale particles and can be calculated using Equation (1). Thus, the $N_{G.B.}$ can be calculated using Equation (4), as shown in Figure 11. We found that the $N_{G.B.}$ has a linear relationship with the $A_{G.B.}$ and is given as follows:

(5) $$N_{G.B.}=2.56E^{-3}\times A_{G.B.}-1.50E^{-4}$$

The $N_{G.B.}$ can be calculated using Equation (5) when the $A_{G.B.}$ is given (when as-cast grain size is known). However, when the as-cast grain size was larger than 100 μm (e.g., alloy A), the $N_{G.B.}$ value was quite small because small areas of grain boundary were rarely involved in the recrystallization nucleation. That is, Equation (5) is satisfied when the as-cast grain size is less than 100 μm. Consequently, given the size and volume fraction of the micro-scale particles and as-cast grain size, the recrystallized grain size ($D_{RX}$) can be estimated using the following equations:

(6) $$N_{RX}=N_{Particle}+N_{G.B.}=\frac{1}{{\left(0.5\times d/f-15\right)}^{3}}+2.56E^{-3}\times A_{G.B.}-1.50E^{-4}$$

(7) $$D_{RX}=\sqrt[{3}]{\frac{1}{N_{RX}}}.$$

The physical meaning and values of different symbols used in the calculation of the grain refinement effects are summarized in Table 4.

### 4.2. Mechanical Properties of Alloy Sheets

#### 4.2.1. Strength

**Figure 12:**
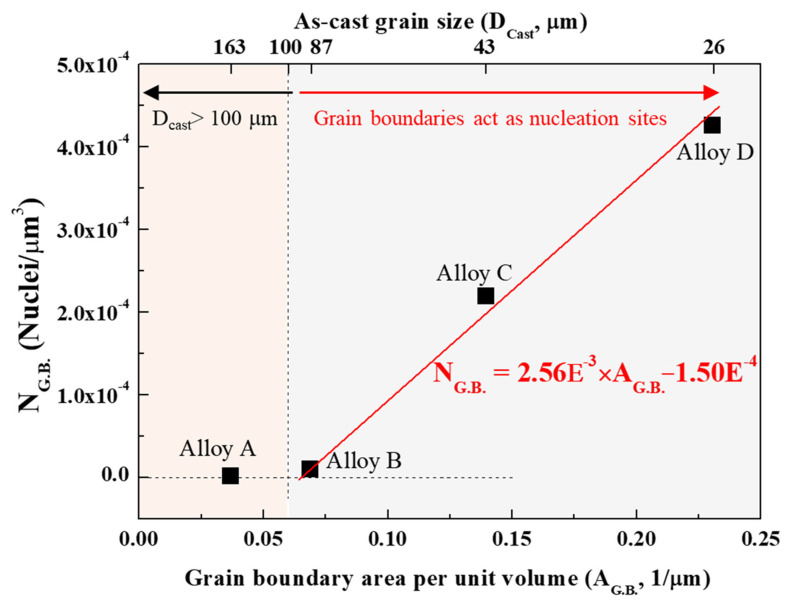
The number of nuclei for recrystallized grains induced by grain boundaries ($N_{G.B.}$) vs. the area of grain boundary per unit volume ($A_{G.B.}$). When the as-cast grain size is less than 100 μm, the $N_{G.B.}$ has a linear relationship with the $A_{G.B.}$ and is given by $N_{G.B.}=2.56E^{-3}\times A_{G.B.}-1.50E^{-4}$.

Table 2 presents the mechanical properties of the recrystallized and aged sheets. The YS of the recrystallized and aged sheets gradually increased without a significant loss in the EL as the average grain size decreased. For the recrystallized and aged sheets, the difference in the YS between alloy sheets A and D was 77.5 and 104.9 MPa, respectively. These strengthening effects can be explained by the grain boundary, particle, and additional precipitate strengthening, as shown in Figure 12. The solid solution strengthening in alloy sheets A, B, C, and D was not considered because the contents of Zn, Mg, and Cu in Al were similar.

**Figure 13:**
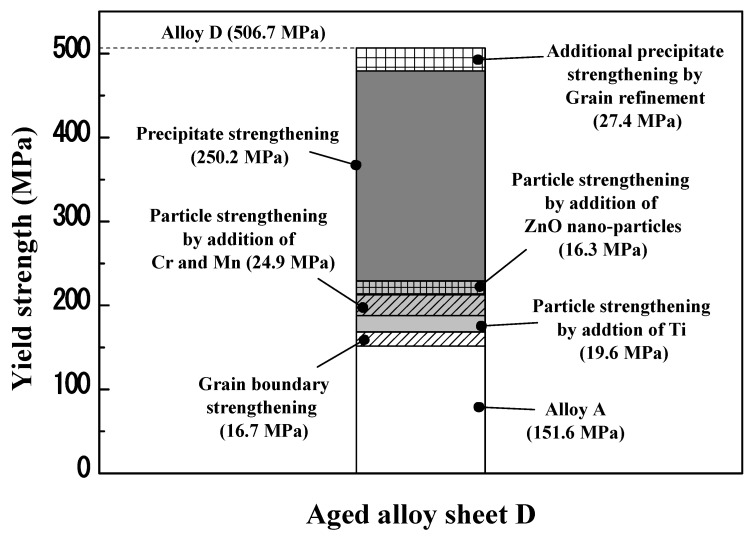
Estimated strength increment for different strengthening mechanisms in aged alloy sheet D compared to aged alloy sheet A. When the average grain size decreases from 54 to 13 μm, the value of $\Delta \sigma_{G.B.}$ is 16.7 MPa. The values of $\Delta \sigma_{Particle}$ contributed by addition of Ti, Cr and Mn, and ZnO nano-particles are 19.6, 24.9, and 16.3 MPa, respectively. The additional precipitate strengthening of 27.4 MPa in alloy D is due to the effect of grain refinement in the recrystallized sheet on the precipitation behavior.

For the recrystallized sheets without natural aging, the strengthening effect can be explained by grain boundary and particle strengthening because there was no precipitate strengthening by the Guinier–Preston (GP) zones, η′- and η-precipitates. In general, the relationship between the YS and the average grain size (*d*) can be calculated by the Hall–Petch equation, as follows:

(8) $$\sigma_{G.B.}=\sigma_{0}+k_{y}\cdot d^{-1/2},$$

where $\sigma_{0}$ and $k_{y}$ represent the friction stress and Hall–Petch slope, respectively. Wert [43] investigated the YS of 7075 alloys with different grain sizes and found that the value of k*_y_* was 120 MPa μm^1/2^. According to the reported k*_y_* value, when the average grain size decreased from 54 to 13 μm, the value of $\Delta \sigma_{G.B.}$ was approximately 16.7 MPa as shown in Figure 13. This is consistent with several studies indicating that the grain refinement in Al–Zn–Mg–Cu alloys cannot significantly improve the strength [5,44]. Therefore, when Ti, Cr and Mn, and ZnO nano-particles are cumulatively added, the YS increments are more affected by particle strengthening than grain boundary strengthening. The particle strengthening ($\Delta \sigma_{Particle}$) can be calculated by subtracting $\Delta \sigma_{G.B.}$ from the total YS increments. The value of $\Delta \sigma_{Particle}$ for the plate-shaped particles containing Mg and Ti was 19.6 MPa, and the value of $\Delta \sigma_{Particle}$ for the round- and rod-shaped particles containing Cr and Mn was 24.9 MPa. The value of $\Delta \sigma_{Particle}$ for the small and large needle-shaped particles was 16.3 MPa. For round-shaped particles containing Cr with an average radius of 34 nm and a volume fraction of 0.13%, the Δσ_Particle value was approximately 14 MPa, which is close to the value reported in a previous study [45].In addition, the particle strengthening increased from 19.0 to 32.9 MPa as the volume fraction of the particles increased by more than 0.5% [46], which is consistent with our study.

For the alloy sheet D aged at 120 °C for 12 h, the strengthening effect can be explained by the grain boundary, particle, and additional precipitate strengthening. It was assumed that the grain boundary and particle strengthening were similar in both recrystallized and aged sheets. This is because the recrystallized grain size and nano-scale particles containing Ti, Cr, and Mn did not show significant changes during the aging treatment at 120 °C due to their high thermal stability [47,48]. In aged sheets A, B, C, and D, the precipitate strengthening by the formation of the GP zones, η′- and η-precipitates, is 250.2, 258.9, 276.0, and 277.6 MPa, respectively. Despite the similar content of Zn, Mg, and Cu, the additional precipitate strengthening of 27.4 MPa in alloy D is due to the effect of recrystallized grain size on the precipitation behavior [49]. The precipitates were more homogeneously formed in fine-grained alloys than in coarse-grained alloys. This is because grain boundaries rich in solute atoms and vacancies are beneficial sites for the nucleation and growth of η-precipitates [50,51]. When the grain size of the recrystallized sheets was large, the total area of grain boundaries per unit volume decreased, and a large amount of solute atoms were severely segregated at local grain boundaries [52]. Therefore, alloy A with large grains exhibited coarser continuous grain boundary precipitates (GBPs), whereas alloy D with relatively small grains exhibited finer discontinuous GBPs [53]. The finer discontinuous GBPs had a significant strengthening effect and better fracture toughness than the coarser continuous GBPs. The coarser continuous GBPs increase the probability of intergranular fracture, which reduces the fracture toughness in Al–Zn–Mg–Cu alloys [54]. Furthermore, Hu et al. [55] reported that the grain refinement influences the precipitation kinetics, leading to the uniform nucleation and growth of dense precipitates in the interior of the grains. Consequently, the grain refinement of the recrystallized sheet not only contributes to grain boundary strengthening but also provides additional precipitation strengthening.

#### 4.2.2. Elongation

The trade-off between strength and elongation is overcome through the grain refinement [5,56]. The grain refinement can delay the formation of large-scale necking by reducing the stress concentration [57]. As shown in Figure 8a,b, the KAM maps confirm that a large amount of stored energy was concentrated at the grain boundaries owing to the piled-up dislocation. For alloy sheet D with smaller grains, the stored strain energy was distributed uniformly over the entire grain boundary, reducing the stress concentration. In contrast, in alloy sheet A with larger grains, the stored strain energy was concentrated at local grain boundaries. This stress concentration induced the formation of microcracks, according to the theoretical mechanism of crack nucleation. In addition, the resistance to crack initiation and propagation can be enhanced by refining the grain size (d). The critical shear stress ($\tau_{f}$) for the crack formation is expressed as follows [58]:

(9) $$\tau_{f}={\left(\frac{2G\gamma_{s}}{d}\right)}^{1/2},$$

where G and $\gamma_{s}$ represent the shear modulus and the surface energy of the crack, respectively. Additionally, the propagation direction repeatedly changes as the crack propagation is limited by grain boundaries, increasing the critical stress ($\tau_{p}$) for crack propagation. The $\tau_{p}$ can be expressed as follows [52]:

(10) $$\tau_{p}={\left(\frac{4E\gamma_{s}}{\pi d}\right)}^{1/2},$$

where *E* represents the elastic modulus. The values of $\tau_{f}$ and $\tau_{p}$ increase as the grain size decreases, indicating that the resistance to crack initiation and propagation can be enhanced by grain refinement.

Furthermore, a large number of activated dislocations induced by the grain boundaries improve the plastic deformation ability, increasing the EL. When the recrystallized grain size decreases from 54 to 13 μm (Figure 3), the area of the grain boundary increases more than four times in three dimensions [40]. Under stress conditions, the grain boundaries can simultaneously serve as nucleation sites and sinks for the dislocations [59,60]. Thus, in the fine-grained sheets with a large grain boundary area, a large number of activated dislocations enhance the plastic deformation ability, leading to an improvement in EL.

## 5. Conclusions

The effects of the as-cast grain size on the formation of recrystallized grains during TMT processes were systematically studied, and the related mechanical properties of the sheets were evaluated. Our findings are as follows:

1. The addition of Ti, Cr and Mn, and ZnO nano-particles significantly reduced the grain size of the Al–Zn–Mg–Cu cast alloys from 163 to 26 μm. As the as-cast grain size decreased from 163 to 26 μm, the recrystallized grain size decreased correspondingly from 54 to 13 μm. This is because the number of nuclei contributed by the micro-scale particles and as-cast grain boundaries increased simultaneously. As the as-cast grain size decreased, the average size of the eutectic particles decreased from 4.4 to 3.0 μm, resulting in an increase in the $N_{Particle}$. Importantly, we found that the $N_{G.B.}$ was proportional to the area of as-cast grain boundaries when the as-cast grain size was smaller than 100 μm (e.g., alloys B, C, and D). As the as-cast grain size decreased, the area of as-cast grain boundaries increased, resulting in an increase in the $N_{G.B.}$. Consequently, given the size and volume fraction of micro-scale particles and the as-cast grain size, the recrystallized grain size can be estimated using the equations proposed in this study.
2. The strengthening effects of the investigated Al–Zn–Mg–Cu sheets can be explained by the grain boundary, particle, and additional precipitate strengthening. The increment in the YS due to grain boundary strengthening was approximately 16.7 MPa when the average grain size decreased from 54 to 13 μm. In addition, the particle strengthening by the addition of Ti, Cr and Mn, and ZnO nano-particles was 19.6, 24.9, and 16.3 MPa, respectively. After the aging treatment, the additional precipitation strengthening in sheet D was 27.4 MPa due to the fine discontinuous GBPs despite similar contents of Zn, Mg, and Cu.
3. The grain refinement delayed the formation of large-scale necking by reducing the stress concentration. The stored strain energy was concentrated at the local grain boundary in the coarse-grained sheets, whereas it was distributed uniformly over the entire grain boundary in the fine-grained sheets. Therefore, the trade-off between strength and elongation was overcome through grain refinement.

## Figures and Tables

**Table 1:** Measured chemical compositions (in wt.%) of the Al–Zn–Mg–Cu cast alloys. Alloy D was obtained by adding 1.0 wt.% ZnO nano-particles to alloy C using techniques for the decomposition of ceramic nano-particles.

Sample	Zn	Mg	Cu	Ti	Cr	Mn	Al	Note
**Alloy A**	6.11	2.74	1.50	-	-	-	Bal.	
**Alloy B**	6.02	2.62	1.41	0.18	-	-		
**Alloy C**	6.14	2.68	1.48	0.20	0.04	0.17		
**Alloy D**	6.36	2.58	1.41	0.19	0.05	0.17		Addition of ZnO nano-particles

**Table 2:** Chemical compositions of the nano-scale particles in Figure 6 measured via EDS (at.%).

Mark	Al	Zn	Mg	Cu	Fe	Cr	Mn	Ti
**S1**	77.59	8.59	11.44	2.38	-	-	-	-
**S2**	84.48	2.01	9.11	0.64	-	-	0.89	2.87
**S3**	83.06	5.37	5.10	4.07	0.34	1.78	0.28	-
**S4**	91.37	3.79	3.39	1.21	0.05	0.05	0.10	0.02
**S5**	75.87	18.05	2.49	3.16	-	0.13	0.27	0.02

**Table 3:** **Average grain size and mechanical properties of recrystallized and aged sheets.** As the grain size of the recrystallized and aged sheets decreased, the YS and UTS increased, without significant loss of EL.

Sample	Recrystallized Sheets				Aged Sheets		
	Grain Size (µm)	YS (MPa)	UTS (MPa)	EL (%)	YS (MPa)	UTS (MPa)	EL (%)
**Alloy A**	54	151.6	317.4	24.3	401.8	525.8	6.4
**Alloy B**	37	174.3	350.4	29.3	433.2	564.8	12.7
**Alloy C**	16	209.5	392.8	25.2	485.5	588.0	12.3
**Alloy D**	13	229.1	421.4	28.4	506.7	606.8	13.8

**Table 4:** Physical meaning and values of different symbols used in the calculation of the grain refinement effect according to as-cast grain size.

Symbol	Meaning	Values	Unit
$D_{Cast}$	As-cast grain size	Measured	μm
$D_{RX}$	Recrystallized grain size	$\sqrt[{3}]{\frac{1}{N_{RX}}}$ [9,15]	μm
$D_{Particle}$	Recrystallized grain size considering only the effect of micro-scale particles	$0.5\times d_{P}/f_{P}-15$	μm
$d_{P}$	The average size of micro-scale particles	Measured	μm
$f_{P}$	The volume fraction of micro-scale particles	Measured	Vol.%
$N_{RX}$	Total number of nuclei for recrystallized grains	$N_{Particle}+N_{G.B.}$	nuclei /μm^3^
$N_{Particle}$	Number of nuclei for recrystallized grains induced by micro-scale particles	$\frac{1}{{D_{Particle}}^{3}}$ [42]	nuclei /μm^3^
$N_{G.B.}$	Number of nuclei for recrystallized grains induced by grain boundaries	$2.56E^{-3}\times A_{G.B.}-1.50E^{-4}$	nuclei /μm^3^
$A_{G.B.}$	Grain boundary area of the cast alloy per unit volume	$6/D_{Cast}$ [40]	μm^2^

## Data Availability

The raw/processed data required to reproduce these findings cannot be shared at this time due to technical or time limitations.
